# Proteomics in myelodysplastic syndromes: towards novel prognostic and diagnostic biomarkers

**DOI:** 10.11613/BM.2026.020504

**Published:** 2026-06-15

**Authors:** Ruđer Novak, Inga Mandac Smoljanović, Lovorka Grgurević

**Affiliations:** 1 Centre for translational and clinical research, Department of Proteomics, School of Medicine, University of Zagreb, Zagreb, Croatia; 2 BIMIS – Biomedical research center Šalata, School of Medicine, University of Zagreb, Zagreb, Croatia; 3 Division of Hematology, Clinical Hospital Merkur, Zagreb, Croatia; 4 Department of Anatomy “Drago Perović”, School of Medicine, University of Zagreb, Zagreb, Croatia

**Keywords:** myelodysplastic neoplasms, acute myeloid leukemia, biomarker, proteomics

## Abstract

Myelodysplastic neoplasms (MDS) are clonal hematopoietic disorders defined by ineffective hematopoiesis, cytopenias, and variable risk of progression to acute myeloid leukemia. Although genomic and epigenomic studies have provided insight into disease pathogenesis, reliable biomarkers for diagnosis and prognosis remain limited. Proteomics offers an important advantage because it reflects the functional protein state and captures post-translational modifications, making it highly relevant for risk assessment and therapy guidance. Recent studies have identified several groups of candidate biomarkers. Kinases and signal transduction proteins such as CAMK1D, PRKCZ, KIT, MAST4, PAK6, PTK7, and NTRK1 are dysregulated in MDS and associated with poor outcomes, immune evasion, and aberrant stem cell signaling. Oncofetal proteins like IGF2BP3 and signaling regulators such as RBP4 further highlight proteomic signatures linked to chemoresistance and subtype specificity. In the transplant setting, immune regulators including CSK, FGR, CRTAM, GP1BA, UBE2N, and STAT1 may serve as predictors of graft rejection and relapse. Cytoskeletal and extracellular matrix proteins such as CEP55, Talin-1, Kindlin-3, Vinculin, THBS1, LRG1, SPARC, SAA1, Clusterin, and PRDM16 underscore the role of bone marrow microenvironmental remodeling and adhesion defects in disease progression. Finally, metabolic enzymes such as LDHA reflect altered energy metabolism and correlate with more aggressive disease biology. Collectively, these proteomic candidates illustrate the complex interplay of signaling, immune regulation, bone microenvironment, and metabolism in MDS. Their validation in clinical cohorts could enable early detection, refined risk stratification, and new therapeutic avenues, positioning proteomics as a central tool in the future management of MDS.

## Graphical abstract

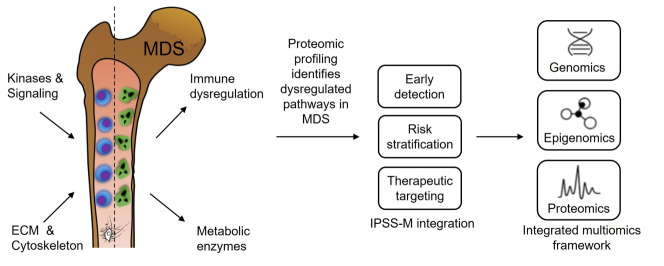

## Highlights

• Myelodysplastic neoplasms are syndrome is defined by cytopenias and ineffective hematopoiesis

• Dysregulated signaling proteins involved in immune evasion are linked to poor outcome

• Bone marrow microenvironment remodeling drives disease progression and adhesion defects

• Proteomics biomarkers may guide therapy and improve patient outcomes

## Myelodysplastic neoplasms

Myelodysplastic neoplasms (MDS) are a group of malignant clonal hematological disorders characterized by ineffective hematopoiesis and dysplasia affecting cell lineages of the bone marrow. It typically presents with nonspecific symptoms related to cytopenias, including fatigue from anemia, infections from neutropenia, and bleeding or bruising from thrombocytopenia. The annual incidence of MDS is approximately 4.9 *per* 100,000. Although it is rare before age 40, it rises sharply after 70, with a median age at diagnosis of 65 years and a slightly higher incidence in men (*1*). Congenital, environmental and life-style factors contribute to the risk of developing MDS. Hereditary bone marrow failure syndromes are well-established predisposing conditions, while environmental risks include benzene exposure from cigarette smoke and occupational exposure to solvents, paints, and pesticides. Epidemiological studies also link higher body mass index (BMI) to increased risk of MDS, with relative risks of 1.15 for overweight, 2.18 for obese adults, and a particularly elevated risk for people with BMI > 35 kg/m^2^ (*2*). The increased risk might stem form obesity-related inflammation, immune dysregulation, and adipocyte-driven alterations of the bone marrow microenvironment (*3*, *4*). Myelodysplastic neoplasms may also arise as a secondary condition from pre-existing hematologic disorders or following cytotoxic chemotherapy and/or radiotherapy. Therapy-related MDS is linked to alkylating agents, topoisomerase II inhibitors and autologous stem cell transplantation (ASCT). It accounts for up to 15% of cases, and carries a markedly increased risk of myeloid neoplasms (*5*, *6*). Elevated MDS incidence has also been observed after solid organ transplantation, likely due to prolonged immunosuppression, with agents such as azathioprine conferring a particularly high risk through impaired DNA repair and chromosomal instability (*7*-*10*).

## Pathogenesis of myelodysplastic neoplasms

Despite extensive study, the exact “cell of origin” in MDS remains unclear. Early MDS is marked by hypercellular bone marrow, coupled with peripheral cytopenias that are driven by increased apoptosis and impaired differentiation of affected cells. This apoptosis may reflect immune responses against MDS antigens, but it also arises from mitochondrial dysfunction and the activation of pro-apoptotic pathways, exacerbated by a raise in pro-inflammatory cytokines. The paradox of high apoptosis in early MDS *versus* reduced apoptosis reported during clonal expansion in advanced disease, suggests a dynamic shift in cellular programs. These insights position apoptosis as both a hallmark of MDS-related bone marrow failure and a potential biomarker and therapeutic target (*11*-*13*).

Recent advances in molecular profiling have reshaped our understanding of MDS pathogenesis, revealing a complex interplay between multiple factors. Genetic and cytogenetic abnormalities influence disease phenotype, progression, and treatment response, thus defining different MDS subtypes and carrying major prognostic value. Cytogenetic lesions occur in roughly half of *de novo* cases, while recurrent mutations that affect RNA splicing, epigenetic regulation, transcription and signaling, are detected in over 90% of cases (*14*).

Myelodysplastic neoplasms incidence rises sharply after age 70, which centers aging in its pathogenesis. Age-related defects in DNA repair, epigenetic regulation, metabolism, and chronic inflammation create a permissive environment for somatic mutations, particularly in *DNMT3A*, *TET2*, and *ASXL1*, that are considered hallmarks of age-related clonal hematopoiesis (*15*, *16*). These processes drive clonal selection and hematopoietic imbalance, providing insight into early MDS evolution and guiding the search for predictive genetic biomarkers (*11*, *12*, *17*). Somatic mutations in genes regulating RNA splicing (*SF3B1, SRSF2, U2AF1*), DNA methylation (*TET2, DNMT3A*), and histone modification (*ASXL1, EZH2*) are common early events in MDS, disrupting gene expression and differentiation to drive clonal hematopoiesis (*18*). Additional mutations in signaling pathways (*NRAS*, *JAK2*), transcription factors (*RUNX1*), or tumor suppressors (*TP53*) promote clonal dominance, genomic instability, and leukemic transformation (*19*, *20*). Through the stepwise accumulation of such genetic and epigenetic alterations that drive clonal expansion, impaired differentiation, and increased blast proliferation, MDS can progress to acute myeloid leukemia (AML) (*21*).

Myelodysplastic neoplasms was classified in 2022 by the World Health Organisation (WHO) 5th Edition, which replaced “syndromes” with “neoplasms” and defined entities based on morphology and specific genetic markers, including MDS with low blasts and isolated chromosome deletion del(5q), MDS with low blasts and *SF3B1* mutation, and MDS with biallelic *TP53* inactivation (*22*). Similarly, the International Consensus Classification (ICC) recognizes the mentioned genetically defined entities, but classifies patients with 10-19% blasts as „MDS/AML“, whereas WHO retains them as MDS with increased blasts-2 (*23*). Both systems rely on morphologic criteria (≥ 10% dysplasia in one or more lineages, blast counts in marrow and blood, and ring sideroblasts) alongside genetic abnormalities to define subtypes (*24*).

Several pre-MDS indolent myeloid disorders have also been recognized: idiopathic cytopenia of undetermined significance (ICUS), idiopathic dysplasia of undetermined significance (IDUS), clonal hematopoiesis of indeterminate potential (CHIP), and clonal cytopenia of undetermined significance (CCUS) (*25*-*27*). Idiopathic cytopenia of undetermined significance involves persistent cytopenia without MDS-related dysplasia or cytogenetic/molecular abnormalities (*17*, *28*, *29*). Idiopathic dysplasia of undetermined significance features morphological dysplasia with minimal or no cytopenia and lacks clonal mutations or cytogenetic changes. Clonal hematopoiesis of indeterminate potential is defined by age-related mutations in genes involved in myeloid malignancies, without cytopenias or neoplastic features. Clonal cytopenia of undetermined significance combines unexplained cytopenia with clonal mutations, often with minimal dysplasia but without MDS-defining abnormalities. These entities provide a valuable model for developing and validating diagnostic and prognostic biomarkers (*11*, *26*-*29*).

## Diagnostic and therapeutic approaches

Given the marked biological heterogeneity of MDS, accurate classification is essential for diagnosis and therapeutic decision-making. Recent WHO and ICC updates integrate morphology and genetic aberrations since cytogenetic or molecular alterations are detectable in approximately 90% of cases. Myelodysplastic neoplasms diagnosis thus requires evidence of dysplastic changes in ≥ 10% of cells in at least one hematopoietic lineage, fewer than 20% myeloblasts in peripheral blood or bone marrow, and presence of clonal genetic abnormalities. However, caution is needed, as many of these mutations also occur in healthy individuals or in related conditions (*21*). Some patients exhibit cytopenias without meeting full MDS criteria, often with stable blood counts for years (*26*). Myelodysplastic neoplasms is further characterized by its inherent risk of progression to AML. The risk was initially stratified by the International Prognostic Scoring System-Revised (IPSS-R) based on marrow blast percentage, cytogenetic risk group, hemoglobin concentration, platelet count, and absolute neutrophil count (*30*). This framework was expanded in 2022 by the International Prognostic Scoring System-Molecular (IPSS-M), by incorporating the mutational status of relevant genes (*i.e*. *TP53, SF3B1, TET2, ASXL1, RUNX1*) (*31*). This refines risk stratification and enables treatment adapted to patient age, comorbidities, and genetic profile, thus improving quality of life and delaying the progression to AML.

Therapeutic options for MDS currently range from conventional therapies, such as hypomethylating agents and lenalidomide, to intensive myeloablative regimens. Although these approaches may induce clinical responses, they fail to fully eradicate MDS stem cells (*32*-*34*). Current research in MDS increasingly focuses on common genetic abberations, like targeting *TP53* using its reactivator Eprenetapopt, or IDH1/2 inhibitors and luspatercept for patients with *SF3B1* mutations (*21*, *35*). Allogeneic hematopoietic stem cell transplantation (HSCT) is the only potentially curative therapy for MDS (*36*, *37*). However, the disease is sustained by diverse and adaptable stem cell populations, which complicates their eradication, and relapse remains the leading cause of treatment failure. Maintenance and pre-emptive therapies following HSCT are, therefore, also areas of active investigation (*38*, *39*). Ultimately, the development of new approaches like digital diagnostics and peripheral blood omics may change future diagnostic practices. Effective management of MDS will likely depend on rational combinations of classical supportive and non-targeted treatments with individualized, mutation-directed therapies (*40*, *41*).

## Protein biomarkers of myelodysplastic neoplasms

As classification systems increasingly integrate molecular features, proteomic profiling offers an opportunity to refine these categories by capturing the functional consequences of genetic lesions and microenvironmental alterations of MDS. This highlights the need to evaluate protein-level biomarkers alongside established genomic markers. Proteomic studies in MDS have utilized several sample types, capturing distinct layers of disease biology. Peripheral blood offers a minimally invasive source suitable for longitudinal monitoring and reflects systemic inflammatory and metabolic alterations. Bone marrow provides a more direct view of the dysplastic niche and microenvironmental remodeling. Finally, stem and progenitor cell-enriched fractions allow the identification of protein signatures specifically associated with clonal hematopoiesis and early leukemogenic events.

Despite significant advances, robust and clinically applicable biomarkers capable of predicting disease course and stratifying high-risk patients remain a major unmet need (*42*-*46*). The purpose of this review is to integrate and evaluate candidate protein biomarkers with potential prognostic relevance and therapeutic value in MDS. These proteins can be categorized according to their predominant biological functions into: kinases and signal transduction molecules, proteins implicated in graft rejection, cytoskeletal and extracellular matrix (ECM) regulators, and metabolic enzymes. Such classification facilitates a clearer understanding of their roles in disease pathogenesis and also highlights potential therapeutic avenues. These molecules were selected *via* a comprehensive literature review and are proposed for future validation of their diagnostic and prognostic utility in MDS.

### 1. Kinases/Signal transduction molecules

Protein kinases have a critical role in cell signaling and regulation. Through phosphorylation, they modify the function, activity, localization, or interactions of their target proteins. Dysregulated kinase activity is proposed as a defining feature of MDS progression and is closely associated with poor prognosis in patients. Individual kinase alterations are well characterized in other cancers (*e.g*., KIT in systemic mastocytosis, neurotrophic tropomyosin kinase receptors fusions in solid tumors, but their collective dysregulation in MDS suggests a unique pathogenic network (*47*, *48*). While individual biomarkers may not be relevant, their collective expression, synthesized into a diagnostic panel, has proven useful for stratifying patients into subgroups based on disease severity. The expression profiles and activity of several kinases has recently been characterized and annotated to MDS patient subgroups using the Kinase Stratification Score (KISS) (*42*). These proteins converge on three significant pathways: immune evasion, stem cell dysregulation and therapeutic resistance. Immune evasion is a hallmark of cancer development. It allows malignant cells to avoid detection by the host immune system, a process that is often orchestrated through dysregulated signaling pathways. Aberrant calcium/calmodulin and Wnt signaling pathway were suggested to be of particular importance to MDS. A notable example is a calcium/calmodulin-dependent kinase (CAMK1D) that modulates the differentiation and apoptosis of haematopoetic cells (*49*). Yao *et al.* associated high expression of CAMK1D in MDS patients with poor overall survival rate, likely due its suppression of caspase-mediated apoptosis, which permits leukemic clone survival. Another member of this signaling pathway is protein kinase C zeta (PRKCZ) whose elevated expression was linked to poor prognosis (*42*). The cluster of Wnt-related kinases were also implicated in the pathology of this disease. Namely, elevated expression of the transmembrane receptor tyrosine kinase KIT appears to further disrupt stem cell signaling and to increase blast counts, thus potentially intersecting functionally with CAMK1D activity in the progression of MDS (*50*). Elevated expression levels of several other Wnt-related kinases have been proposed as potential prognostic biomarkers within the KISS model, although their mechanisms of contribution to MDS pathogenesis are largely unknown. In particular, overexpression of microtubule-associated serine/threonine kinase 4 (MAST4), p21-activated kinase 6 (PAK6), and protein tyrosine kinase 7 (PTK7) have been linked to adverse outcomes. Moreover, while mutations in neurotrophic receptor tyrosine kinase 1 (*NTRK1*) are commonly associated with solid tumours, they have also been correlated with poor overall survival (*42*). *NTRK1* mutations in MDS often result in constitutive kinase activation, analogous to gain-of-function mutations in solid tumours, implicating a shared “hyperactive signaling” phenotype despite divergent mutational spectra (*51*). Functionally, patients classified as KISS-high exhibit concurrent overexpression of all seven kinases. This implies that the kinases are not in direct biochemical interaction and that they act in additive manner in pathogenesis of MDS.

Insulin-like growth factor 2 mRNA-binding protein 3 (IGF2BP3) exerts broadly detrimental effects on patient prognosis. This oncofetal protein, typically low in healthy adults, is frequently re-expressed in cancers. In MDS and AML patients, elevated plasma concentrations of IGF2BP3 are associated with chemotherapy resistance and reduced survival. These effects are thought to result from the stabilization of survival-promoting mRNAs, including modulators of the PI3K-AKT and MAPK pathways, thereby inhibiting apoptosis (*52*).

Retinol-Binding Protein 4 (RBP4) is increasingly recognized not only as a transporter of retinol but also as a signaling molecule that modulates metabolic and inflammatory pathways. Through its endocrine-like effects, RBP4 can influence cellular communication and immune responses, placing it among proteins with signaling functions. The RBP4 is a blood transporter of retinol (vitamin A), with a role in multiple metabolic and inflammatory pathways (*53*). Elevated concentrations of RBP4 expression have been observed in certain subtypes of MDS, such as refractory anemia with excess blasts-1 (*54*). However, RBP4 concentrations remain normal in MDS subtype, anemia with ringed sideroblasts, suggesting that RBP4 may serve as a potential biomarker specific to certain MDS subgroups (*55*).

Kinases are among the most important drug targets in the human proteome, with a long history of being targeted for an expanding range conditions (*56*). Understanding the kinase activation patterns driving MDS is a crucial insight into the mechanisms underlying disease progression. Targeting these dysregulated pathways holds the potential to modulate ineffective hematopoiesis or reduce malignant cell survival. With the exception of PTK7, for which inhibitory strategies have shown only limited success in phase 1 clinical trials, inhibitors of other potential immune evasion mediators have yet to be evaluated in the context of MDS (Table 1) (*51*, *57*-*61*). Table 1 summarizes the most frequently reported proteomic biomarkers in MDS. To aid interpretation, the column ‘Validation potential’ reflects an assessment based on several criteria: (i) reproducibility across independent cohorts; (ii) availability of orthogonal confirmation (*e.g*., ELISA, flow cytometry, targeted MS); (iii) demonstrated association with clinically relevant endpoints such as survival, progression, or treatment response; and (iv) feasibility of measurement in routine clinical samples such as peripheral blood, bone marrow plasma, or CD34+ fractions. These criteria collectively indicate the likelihood that each biomarker could progress toward clinical validation.

**Table 1:** Candidate protein biomarkers in myelodysplastic neoplasms (MDS) with potential prognostic and therapeutic relevance.

**Molecule**	**Pathway/Category**	**Reported effect in MDS**	**Prognostic implication**	**Validation potential**	**Reference**
CAMK1D	Calcium/calmodulin signaling kinase	Suppresses caspase-mediated apoptosis, modulates hematopoietic differentiation	High expression linked to poor overall survival	Strong candidate biomarker; warrants functional and clinical validation	(*42*, *49*)
PRKCZ	Protein kinase C family	Dysregulated expression contributes to immune evasion	Associated with poor prognosis	Promising, requires further mechanistic studies	(*42*)
KIT	Receptor tyrosine kinase (Wnt-related)	Alters stem cell signaling, increases blast counts	Overexpression linked to disease progression	Targetable kinase, potential therapeutic biomarker	(*50*)
MAST4	Serine/threonine kinase (Wnt-related)	Mechanism unclear, implicated *via* KISS model	Overexpression associated with adverse outcome	Candidate for exploratory validation	(*42*)
PAK6	Serine/threonine kinase (Wnt-related)	Mechanism unclear, implicated *via* KISS model	Overexpression associated with adverse outcome	Candidate for exploratory validation	(*42*, *61*)
PTK7	Pseudokinase, Wnt signaling modulator	Implicated in MDS *via* KISS model; limited therapeutic inhibition tested	High expression linked to poor prognosis	Limited clinical trial data; further validation needed	(*42*, *57*)
NTRK1	Neurotrophic receptor tyrosine kinase	Activating mutations cause constitutive signaling	Mutations correlated with reduced survival	Potential shared oncogenic driver; promising biomarker	(*42*, *51*)
IGF2BP3	RNA-binding oncofetal protein	Stabilizes pro-survival mRNAs (PI3K-AKT, MAPK pathways); promotes chemoresistance	Elevated concentrations linked to reduced survival and therapy resistance	Strong candidate for clinical validation	(*52*)
RBP4	Retinol-binding protein, signaling modulator	Alters metabolic and inflammatory signaling; subtype-specific expression	Elevated in RAEB-1 but not in RARS; potential subgroup biomarker	Promising subtype-specific biomarker for stratification	(*53*-*55*)
For each candidate biomarker, it is crucial to distinguish whether clinical implementation would require detection of somatic mutations (*e.g*., NTRK1) or quantification of protein overexpression (*e.g*., KIT, PTK7), as the analytical platforms and clinical significance differ accordingly.					

### 2. Proteins related to graft rejection

Graft rejection is a transplant failure in which the recipient’s immune system attacks the transplanted bone marrow or blood-forming cells, preventing engraftment and recovery. Disruption of the kinase signaling pathways can trigger dysregulated innate and adaptive immune responses. This imbalance promotes inflammation and immune cell infiltration, processes that can ultimately lead to graft rejection. This is of high importance to MDS since HSCT is the only curative option currently available for patients. Therefore, identifying proteins that predict allograft rejection at an early stage of treatment is of great importance for monitoring graft function and assessing relapse risk. Several of these candidate proteins converge on common signalling pathways like NF-κB, and Src kinase pathways implicated in inflammatory signaling and immune cell activation (*62*, *63*).

A likely candidate is C-terminal Src kinase (CSK), which regulates immune homeostasis by preventing the overactivation of inflammatory pathways. Elevated CSK was associated with MDS by post-transplant immune activity and is potentially predictive of disease relapse (*46*). CSK is a known regulator that can control the activity of tyrosine-protein kinase Fgr (FGR), a non-receptor tyrosine kinase primarily expressed in hematopoietic cells (*64*). In physiologic conditions, FGR expression is largely restricted to macrophage subsets; however, during inflammation, it is produced by most hematopoietic cell types. As FGR regulates inflammatory cytokine secretion and immune cell activation, the combined activity of these two modulators could exacerbate the chronic immune dysregulation and ineffective hematopoiesis characteristic of MDS (*46*, *65*). Similarly, dysregulation of Lck/yes-related novel (LYN) kinase in hematopoietic cells has been reported in chronic myelomonocytic leukemia and AML (*66*).

Graft rejection is largely driven by cytotoxic T cells that recognize donor antigens, trigger inflammation, and destroy graft tissue. This process is amplified by class I-restricted T cell-associated molecule (CRTAM), which is expressed on activated cytotoxic immune cells and is overexpressed in patients with MDS. Its overexpression could contribute to enhanced graft rejection and disease relapse. Glycoprotein Ib alpha chain (GP1BA) is a component of the platelet receptor complex von Willebrand factor that is essential for hemostasis and vascular immunity (*46*). Although *GP1BA* is not mutated in MDS, the characteristic dysfunction of megakaryocytes and abnormal platelet activation that present in MDS patients, may reflect its altered levels.

Finally, ubiquitin-conjugating enzyme E2N (UBE2N) mediates the attachment of ubiquitin to target proteins, a key mechanism regulating NF-κB-dependent DNA repair (*67*). Unlike the previously discussed immune modulators, UBE2N primarily influences malignant cell survival. It is overexpressed in leukemic stem and progenitor cells in both MDS and AML, where it supports the maintenance and persistence of these malignant cell populations (*68*). The roles of other immune modulators, such as oxidized LDL receptor 1 (OLR1), S100 calcium-binding protein A12 (S100A12), and signal transducer and activator of transcription 1 (STAT1), have also been implicated in post-transplant complications, though their contributions require further investigation (*46*).

Similar to therapeutic approaches linked to immune evasion, efforts to target graft rejection-related mediators or their pathways in MDS remain in the earliest stages of development. For instance, modulating STAT1 activity is being tested in various disease settings such as myelofibrosis, myeloproliferative neoplasms, thrombocytopenia, and hematologic malignancies, including MDS (*69*-*73*). Such inhibitors may potentially help restore normal hematopoiesis by dampening the overactive JAK-STAT signaling observed in MDS, offering an altrenative therapeutic alley. Similarly, inhibition of UBE2N has been shown to suppress the function and viability of MDS/AML cell lines as well as patient derived samples (*74*, *75*).

**Figure 1:**
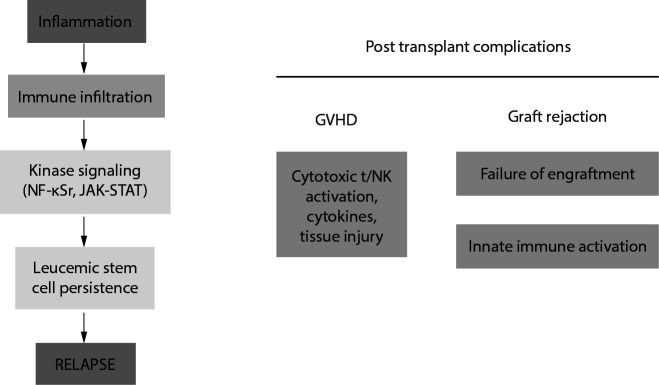
Schematic representation of post-transplant mechanisms in MDS, illustrating the distinction between relapse biology and immunologic transplant complications. Chronic inflammation and immune infiltration activate dysregulated kinase pathways (NF-κB, Src, JAK–STAT), which support leukemic stem cell persistence and ultimately lead to post-transplant relapse. In contrast, graft-*versus*-host disease (GVHD) arises from alloimmune activation of cytotoxic T and NK cells, exemplified by CRTAM-mediated effector responses. Graft rejection reflects failure of donor engraftment, primarily driven by host innate immune activation. These pathways represent biologically and clinically distinct processes, and only the kinase-driven axis contributes to malignant relapse rather than GVHD or graft rejection.

Beyond these, multiple other conceptually promising mediators represent druggable targets, however most evidence is limited to early-stage studies or to different disease contexts and their clinical relevance in MDS is yet to be established (Figure 1) (*76*-*78*).

### 3. Bone marrow microenvironment and cytoskeletal/ECM regulators

The bone marrow cytoskeleton orchestrates the regulation of cell morphology, migratory capacity, intracellular signaling, and mitotic division. Several proteins related to impaired cytoskeletal dysfunction have thus emerged as promising therapeutic targets in acute leukemias (*79*). Centrosomal Protein 55 (CEP55) is a cell division modulator whose overexpression is considered to promote tumorigenesis (*80*). Talin-1 (TLN1) is a cytoskeletal protein that plays a role in linking integrins to the actin cytoskeleton, facilitating cell adhesion and migration (*81*). The concentrations of both proteins were elevated in bone marrow plasma of patients with MDS, particularly those with ring sideroblasts. Indeed, increased CEP55 expression has been associated with abnormal karyotypes which strengthens it as a potential for chromosomal instability (*43*). Kindlin-3 has strengthened the understanding of problematic leukocyte adhesion in AML. Its epigenetic suppression (hypermethylation) leads to dysfunctional blood cell adhesion and cell viability in both MDS and AML, potentially exacerbating the bone marrow failure observed in MDS (*82*). Furthermore, kindlin-3 deficiency may contribute to the defective hematopoietic microenvironment of MDS by disrupting integrin signaling. Its mutations may overlap with inflammatory mechanisms that involved in the MDS pathogenesis (*83*). Vinculin is another cytoskeletal modulator involved in cell adhesion and mechano-transduction whose expression levels are altered in MDS. Reduced expression of vinculin in peripheral blood mononuclear cells from MDS patients may contribute to impaired cell adhesion, thereby disrupting hematopoiesis - a hallmark of the disease (*84*). Additionally, its reduced expression in plateletes derived from MDS patients may impair integrin αIIbβ3 activation, resulting in defective platelet aggregation and contributing to the bleeding complications commonly observed in MDS patients (*85*). Finally, increased levels of posttranslational modifications of vinculin have also been suggested as promising cadidate diagnostic biomarkers (*86*).

**Figure 2:**
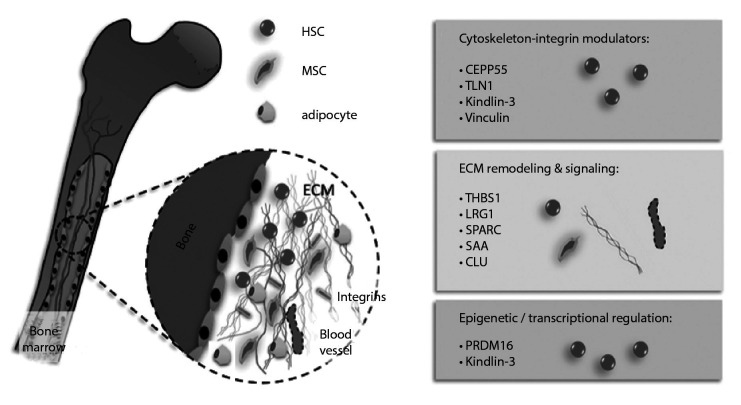
Schematic representation of the bone marrow microenvironment showing cytoskeletal regulators (CEP55, Talin-1, Kindlin-3, Vinculin) and ECM proteins (THBS1, LRG1, SPARC, SAA1, Clusterin), together with PRDM16, illustrating disrupted adhesion, signaling, and remodeling processes driving MDS/AML progression. ECM - extracellular matrix. HSC - hematopoietic stem cell. MSC - mesenchymal stem cell.

The cytoskeleton is in constant crosstalk with the ECM, coordinating adhesion, signaling, and microenvironmental remodeling (*87*). Low concentrations of thrombospondin-1 (THBS1), an ECM glycoprotein that plays a role in angiogenesis, apoptosis and tumor progression have been observed in patients with MDS. Those exhibiting reduced THBS1 expression tend to have shorter overall survival, suggesting its potential utility as a prognostic biomarker (*88*). Pathological angiogenesis has also been associated with leucine-rich α-2-glycoprotein 1 (LRG1), a glycoprotein primarily secreted by hepatocytes and neutrophils. Elevated plasma concentrations of LRG1 in MDS patients are believed to influence the bone marrow microenvironment through its expression and secretion from neutrophil granules. LRG1 promotes aberrant angiogenesis by modulating TGF-β signaling, resulting in the formation of immature and dysfunctional blood vessels (*44*, *89*). Another important mediator of ECM remodelling is secreted protein acidic and rich in cysteine (SPARC, osteonectin). Implicated in several hematologic malignancies, particularly MDS, it is of growing interest due to its dual functions as both a tumor suppressor and tumor promotor. *SPARC* is located on chromosome 5q31.3-q32, a region frequently deleted in MDS, particularly in the 5q-syndrome, a distinct subtype of MDS defined by the deletion of chromosome 5q which has a more favourable prognosis. Similarly, the transcription factor PR domain containing 16 (PRDM16) has recently been implicated in hematologic malignancies. The *PRDM16* gene is located on chromosome 1p36, a region frequently involved in chromosomal translocations in patients with MDS and AML. Translocations of this gene are linked to its overexpression, which is associated with poor prognosis in affected patients (*90*). Secreted by the bone marrow stroma microenvironment, serum amyloid A1 (SAA1) is known to regulate the ECM through matrix metalloprotineases (*91*). Emerging evidence also implicates SAA1 in the pathogenesis of both MDS and AML (*92*). It has been shown to promote the proliferation of malignant clones in these disorders, regardless of cytogenetic or mutational profiles. Targeting SAA1 using IL-6 inhibitors or small peptides could mitigate clonal expansion in MDS (*93*, *94*). Aditionally, as elevated concentrations of SAA1 were correlated with increased disease severity in MDS and its progression to AML, it could have potential as a biomarker for disease monitoring (Figure 2).

One of the most prominent chaperons of the ECM is clusterin. Overexpression of secreted clusterin (CLU) has been linked to resistance to chemotherapy and radiotherapy in various types of cancer (*95*). In patients with MDS, CLU proteoforms in plasma-derived exosomes were compared to “free” plasma CLU concentrations. The observed altered distribution of CLU proteoforms suggests their potential utility as a biomarker for MDS progression (*45*).

### 4. Metabolic enzymes

Increased activity of lactate dehydrogenase A (LDHA) in MDS patients, as indicated by their elevated plasma LDH, may serve as a potential prognostic factor (*96*). LDHA is a glycolytic enzyme that catalyzes the conversion of pyruvate to lactate, thereby sustaining glycolytic flux and supporting energy production under hypoxic conditions. The involvement of this enzyme in tumorigenesis is well-established, reflecting its importance in the metabolic reprogramming of malignant cells (*97*). A wide array of LDHA inhibitors, ranging from synthetic molecules and natural compounds to antisense oligonucleotides, RNA interference, and N-terminal mimetic peptides, are currently under investigation in various cancers (*98*-*100*). However, their role in MDS remains uncertain.

In conclusion, a deeper understanding of the cellular composition of the normal, dysplastic, and leukemic marrow niche, as well as the complex network of intercellular interactions, is expected to provide novel insights into therapeutic approaches in the coming years.

Genomics is an integral part of routine diagnostic and prognostic assessment in MDS. However, despite advances in genomic profiling, MDS remains a therapeutic challenge due to its striking molecular heterogeneity, with patients exhibiting diverse mutational landscapes, epigenetic dysregulation, and metabolic adaptations. Future management will rely increasingly on strategies that integrate disease pathophysiology, biology, and their molecular underpinnings. Proteomics enables the identification of disease-specific protein expression patterns and post-translational modifications, and can uncover novel biomarkers of diagnosis, prognosis, and therapeutic response. Integration of clinical proteomic data into existing diagnostic frameworks may thus lead to improved molecular classification, optimized therapeutic decision-making, and the development of protein-targeted therapies in MDS (*46*).

## Data Availability

No data was generated during this study, so data sharing statement is not applicable to this article.
